# Optimal antithrombotic therapy after transcatheter aortic valve replacement: a comprehensive review

**DOI:** 10.3389/fcvm.2025.1528071

**Published:** 2025-03-10

**Authors:** Nguyen Van Thai Thanh, Myeong-Ki Hong, Young-Guk Ko

**Affiliations:** ^1^ Department of Cardiovascular Surgery, University of Medicine and Pharmacy, Ho Chi Minh City, Vietnam; ^2^ Severance Cardiovascular Hospital, Yonsei University College of Medicine, Seoul, Republic of Korea

**Keywords:** aortic valve stenosis, transcatheter aortic valve replacement, thrombosis, antithrombotic agents, hemorrhage

## Abstract

Transcatheter aortic valve replacement (TAVR) has become a leading treatment for aortic stenosis, but managing thromboembolic and bleeding risks post-procedure remains challenging. This review examines current evidence on antithrombotic therapy after TAVR. Subclinical leaflet thrombosis is observed in 10%–20% of patients, though its clinical significance remains uncertain. Clinical valve thrombosis is rare. Current guidelines favor single antiplatelet therapy for patients without indications for long-term anticoagulation, as dual antiplatelet therapy increases bleeding risk without improving outcomes. For patients requiring long-term anticoagulation, monotherapy with direct oral anticoagulants or vitamin K antagonists is recommended to minimize bleeding. Ongoing trials aim to clarify optimal antithrombotic regimens and strategies for preventing subclinical leaflet thrombosis. Individualized therapy based on patient risk profiles is likely needed to improve the efficacy and safety of antithrombotic treatment post-TAVR.

## 1 Introduction

Aortic stenosis (AS) is one of the most prevalent valvular heart diseases, especially among older adults, and is associated with significant morbidity and mortality if left untreated. Traditionally, surgical aortic valve replacement has been the standard treatment for severe symptomatic AS. However, the advent of transcatheter aortic valve replacement (TAVR) has revolutionized the treatment landscape, offering a minimally invasive alternative with favorable outcomes, particularly for patients at high or prohibitive surgical risk (1, 2). Since its introduction by Cribier et al. in 2002, the use of TAVR has expanded to include lower-risk populations because of technical advances, reduced complications, and favorable outcomes (3). However, the risk of thromboembolic events, myocardial infarction (MI), and both subclinical and clinical valve thrombosis remain critical issues requiring optimal postprocedural management. Balancing the prevention of thromboembolic events against the risk of bleeding is central to managing patients after TAVR (4, 5). Selection of appropriate antithrombotic therapy, including single antiplatelet therapy (SAPT), dual antiplatelet therapy (DAPT), or anticoagulation, remains a subject of debate. This review comprehensively assesses current evidence in the field, focusing on key trials and clinical recommendations to provide insights into the optimal antithrombotic regimen after TAVR.

## 2 Need for antithrombotic therapy after TAVR

Patients undergoing TAVR have an increased risk of thrombotic and bleeding events in the periprocedural period and during long-term follow-up post TAVR because of their older age and comorbidities (4, 5). The possibility of cardiovascular embolic events, such as stroke and MI, and valve thrombosis require the use of preventive antithrombotic therapy after TAVR. However, the older and often frail patient population undergoing TAVR is also at high risk for bleeding, which complicates the choice of therapy. Antithrombotic regimens must be tailored to individual risk profiles that consider various factors, including the presence of atrial fibrillation (AF), coronary artery disease, and previous stroke.

### 2.1 Cardiovascular embolic events

#### 2.1.1 Stroke and transient ischemic attack

Stroke remains a significant complication post TAVR, even though 30-day stroke rates have decreased from 4∼6% in early trials to below 1% in recent studies including PARTNER 3 (6–13). Furthermore, the risk of stroke persists beyond the periprocedural period, with rates ranging between 0.2% and 7.8% at 1 year after TAVR. The mechanisms contributing to periprocedural and delayed strokes differ. Periprocedural strokes are often related to embolization of calcified aortic debris during valve deployment. Although cerebral embolic protection devices may appear to reduce disabling stroke in the PROTECTED TAVR trial, none of the randomized controlled trials, including PROTECTED TAVR, SENTINEL, and REFLECT II, have demonstrated a significant reduction in cerebral ischemic events with these devices, and their adoption remains limited (14–17). Post-TAVR strokes are often associated with new-onset AF or valve-related thrombus formation. Subclinical leaflet thrombosis has been observed in up to 22% of patients undergoing TAVR and is associated with a more than 3-fold increase in stroke risk (18, 19).

#### 2.1.2 Myocardial infarction

Though less common than stroke, MI can also occur during or after TAVR. The risk of MI associated with TAVR ranges from 0% to 2.8% at 30 days and from 0.4% to 3.5% at 1 year (6–13). MI is more likely in patients with preexisting coronary artery disease and those undergoing valve-in-valve procedures, which may result in obstruction of the coronary ostia by the implanted prosthetic valve. Postprocedural coronary ischemia can also result from impaired coronary flow dynamics secondary to interactions between the prosthetic valve and the native aortic anatomy. In rare instances, MI may be caused by coronary embolism. Nonetheless, coronary artery disease is a very common comorbidity in patients undergoing TAVR, and MI is often the result of disease progression.

### 2.2 Valve thrombosis

**Figure 1:**
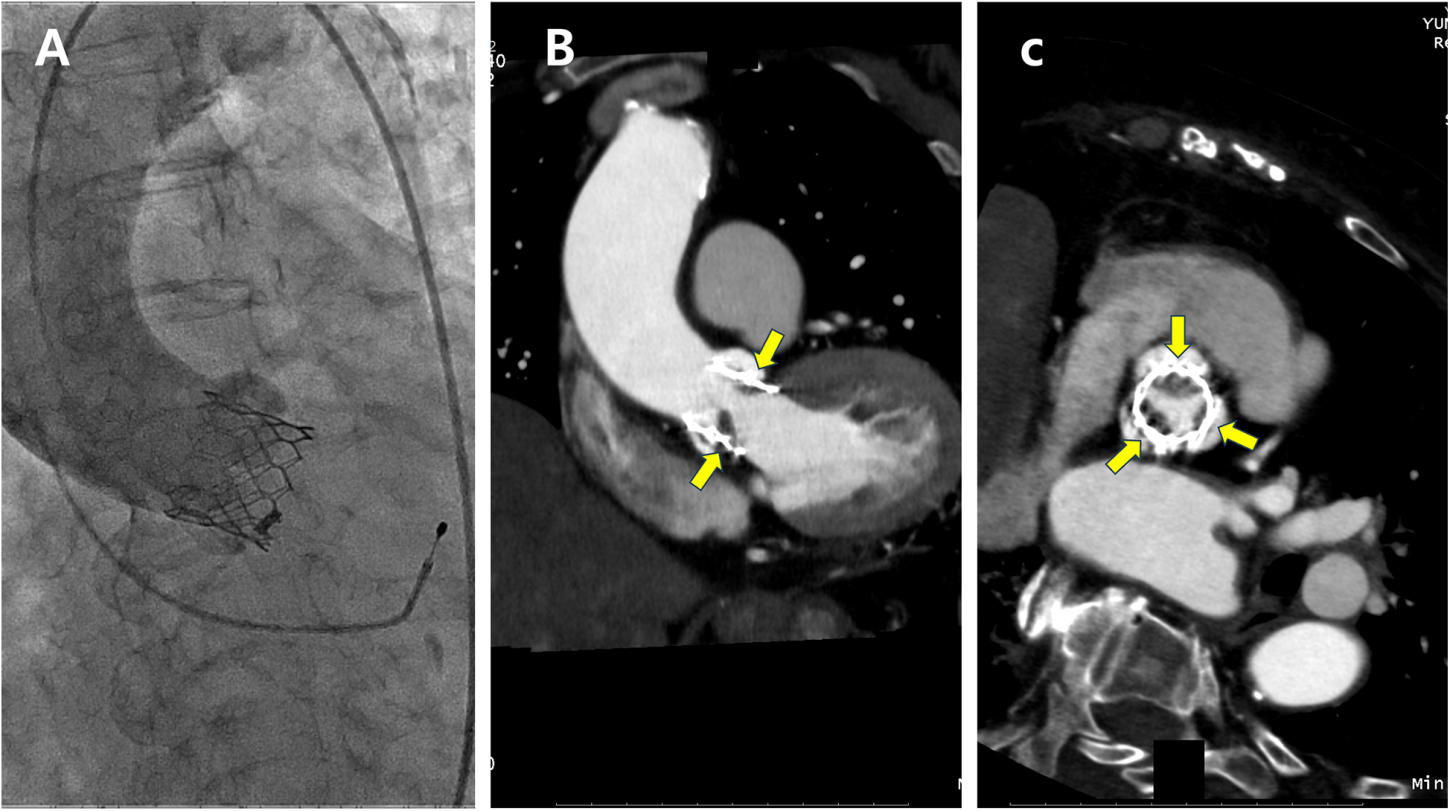
A patient with subclinical leaflet thrombosis detected on computed tomography (CT) at 3-month follow-up. **(A)** Aortography immediately after transcatheter aortic valve replacement. **(B,C)** Implanted aortic valve with hypoattenuated leaflet thickening (yellow arrows) on CT suggestive of leaflet thrombosis (**B**, longitudinal view; **C**, short axis view).

Valve thrombosis encompasses a spectrum ranging from subclinical leaflet thrombosis to clinically apparent valve thrombosis (20). Subclinical leaflet thrombosis is characterized by hypoattenuated leaflet thickening on computed tomography (CT) (Figure 1) and may progress to reduced leaflet motion. Although subclinical leaflet thrombosis is detected in 10%–20% of patients within the first year after TAVR, clinical valve thrombosis occurs much less frequently, with an incidence of approximately 1.2% (20–22).

The exact mechanisms leading to valve thrombosis following TAVR are not fully understood. However, factors such as hypercoagulability at the bioprosthetic surface, leaflet surface damage during device deployment, and blood flow disturbances around the valve may contribute to thrombus formation (20). Subclinical leaflet thrombosis may progress over time to clinical valve thrombosis, which can manifest as valve dysfunction, with an increased transvalvular gradient, overt heart failure, or thromboembolic events (23). However, the clinical significance of subclinical leaflet thrombosis remains uncertain. Several meta-analyses reported conflicting conclusions regarding whether subclinical leaflet thrombosis or reduced leaflet motion is associated with an increased risk of stroke or structural valve deterioration (18, 22, 24–26).

### 2.3 Preexisting and new-onset atrial fibrillation

Atrial fibrillation (AF) and aortic stenosis (AS) share common risk factors, such as advanced age and hypertension (27). AS further increases the risk of AF by elevating pressure overload in the left ventricle and left atrium. AF is observed in 33%–44% of patients undergoing TAVR, reflecting the high-risk profile of this population (28). The prevalence of new-onset AF after TAVR ranges from 6.8% to 9.9%, depending on the study population (29, 30). Risk factors for developing new-onset AF include higher Society of Thoracic Surgeons score, transapical access, pulmonary hypertension, chronic kidney disease, peripheral vascular disease, and severe mitral regurgitation (30). Hemodynamic instability, myocardial injury during the procedure, and the subsequent systemic inflammatory response are thought to contribute to its development during and after TAVR (29, 30).

Both preexisting and new-onset AF are associated with increased cardiovascular complications, including stroke, mortality, and bleeding (29). Preexisting AF primarily increases the risk of late stroke and mortality, while new-onset AF is linked to higher rates of stroke, mortality, and bleeding in the early phase (within 30 days) following TAVR (27, 28, 30). However, the impact of new-onset AF on long-term outcomes, such as late stroke and mortality, remains inconsistent. Although early new-onset AF resolves in approximately 83% of cases, anticoagulation therapy is often underutilized in these patients, leaving them at an elevated risk of cardiovascular embolic events (31). Current guidelines do not specifically address monitoring for new-onset AF or recommend antithrombotic therapy tailored to the type of AF. This highlights an unmet need for individualized antithrombotic strategies in patients with AF undergoing TAVR.

Furthermore, left atrial appendage (LAA) occlusion may be a viable option for patients with AF undergoing TAVR, particularly those at high risk of bleeding. A recent multicenter randomized trial compared concomitant TAVR with LAA occlusion to TAVR combined with medical therapy in patients with AF (32). This study found that the combined procedures were safe and non-inferior to TAVR with medical therapy alone. However, the potential benefits of such combined procedures require further investigation in future clinical trials.

## 3 Bleeding risk after TAVR

Bleeding is a common and serious complication following TAVR, with major bleeding events occurring in 2.2%–41.7% of patients within 30 days and in 2.8%–46.1% within 1 year, depending on the patient's bleeding risk and the valve system used (6–13, 33). According to the recent CENTER 2 trial, a pooled, large-scale patient-level database from 10 clinical trials, the major bleeding rate decreased from 11.95 in 2007–2010 to 5.1% in 2019–2022 (34). Early bleeding (within the first 30 days post procedure) accounts for the majority of bleeding events after TAVR and is often related to procedural and technical factors, such as access site complications. The introduction of smaller delivery systems and the increased use of transfemoral access has led to a reduced incidence of major early bleeding, although the risk remains substantial, especially in patients with a high bleeding risk. Later bleeding events (beyond the first 30 days post TAVR) are typically unrelated to the access site and reflect the combined effects of antithrombotic therapy and patient-related factors.

Bleeding risk in patients undergoing TAVR is influenced by multiple factors, including patient age, frailty, and comorbidities (e.g., chronic kidney disease, anemia) as well as the type and duration of antithrombotic therapy. Older adults with frailty or significant comorbidities are especially vulnerable to gastrointestinal or neurologic bleeding and therefore require close monitoring. Patients with intermediate and high surgical risk experience higher rates of major bleeding compared to those with low surgical risk (34). However, surgical risk scores, such as the Society of Thoracic Surgeons (STS) predicted risk for mortality and the European System for Cardiac Operative Risk Evaluation (EuroSCORE) II, do not reliably correlate with bleeding risks or clinical outcomes in TAVR patients (35). Recently, the Valve Academic Research Consortium for High Bleeding Risk (VARC-HBR) has established specific criteria to identify patients at high risk for bleeding during and after TAVR (33, 36). According to these criteria, over 90% of TAVR patients are considered high bleeding risk. For these individuals, aggressive antithrombotic therapy can result in life-threatening bleeding, emphasizing the need for careful risk stratification and tailored therapeutic strategies. The incorporation of additional bleeding risk categories, such as moderate, high, and very high, could facilitate an individualized, risk-based approach to minimize bleeding complications while optimizing patient outcomes (37).

## 4 Recent trials regarding antithrombotic therapy after TAVR

### 4.1 Patients with no indications for anticoagulation

#### 4.1.1 Dual vs. single antiplatelet therapy

Based on clinical practice after percutaneous coronary intervention, antithrombotic therapy after TAVR initially consisted of 1–6 months of DAPT, followed by lifelong SAPT with aspirin. Two randomized controlled trials evaluated the benefits and risks of SAPT vs. DAPT after TAVR in patients with no indications for anticoagulation. The ARTE (Aspirin Versus Aspirin and Clopidogrel Following Transcatheter Aortic Valve Implantation) trial was the first study comparing SAPT (aspirin) with DAPT (aspirin plus clopidogrel) in these patients (Table 1) (38). The primary endpoint was the composite of death, MI, stroke or transient ischemic attack, or major or life-threatening bleeding (according to VARC-2 definitions) within the first 3 months post TAVR. The study was stopped prematurely after enrolling 222 participants (74% of the planned cohort) because of slow recruitment and lack of continued financial support. The primary composite endpoint tended to occur more frequently in the DAPT group than in the SAPT group at 3-month follow-up, although the difference was not statistically significant (15.3% vs. 7.2%, *p* = 0.065). When each outcome was evaluated separately, the rates of all thromboembolic events were similar between groups, but the rate of major or life-threatening bleeding was significantly higher in the DAPT group than in the SAPT group (10.8% vs. 3.6%, *p* = 0.038).

**Table 1:** Major outcomes of randomized clinical trials of antithrombotic therapy after transcatheter aortic valve replacement for patients with or without indications for chronic anticoagulation.

Trial (reference), year (no. of subjects)	Test group	Control group	Follow-up	Primary endpoint	Bleeding
Patients with no indications for anticoagulation					
ARTE (38), 2017 (*n* = 222)	Aspirin + clopidogrel	Aspirin	3 months	Composite of death, MI, stroke, TIA, or major/life-threatening bleeding (15.3% vs. 7.2%, *p* = 0.065)	Major or life-threatening bleeding events (10.8% vs. 3.6%, *p* = 0.038)
POPular TAVI cohort A (39), 2020 (*n* = 665)	Aspirin	Aspirin + clopidogrel	1 year	Two primary endpoints: all bleeding (15.1% vs. 26.6%, *p* = 0.001); non–procedure-related bleeding (15.1% vs. 24.9%, *p* = 0.005)	Primary endpoints
GALILEO (46), 2020 (*n* = 1,644)	Rivaroxaban 10 mg (+ aspirin for 3 months)	Aspirin 75–100 mg (+ clopidogrel for 3 months)	17 months	Death or thromboembolic event (9.8 vs.7.2 per 100 person-years, *p* = 0.04)	Major, life-threatening, or disabling bleeding (4.3 vs. 2.8 per 100 person-years, *p* = 0.08)
ATLANTIS stratum 2 (47), 2022 (*n* = 1,049)	Apixaban 5 mg bid	Aspirin and/or clopidogrel	1 year	Composite of death, MI, stroke or TIA, non-CNS embolism, pulmonary embolism, intracardiac or valve thrombosis, DVT, or life-threatening, disabling, or major bleeding (16.9% vs. 19.3%, *p* = NS)	Life-threatening, disabling, or major bleeding (7.8% vs. 7.3%, *p* = NS)
ADAPT-TAVR (48), 2022 (*n* = 229)	Edoxaban 60 mg	Aspirin + clopidogrel	6 months	Incidence of valve leaflet thrombosis detected on 4D-CT imaging (9.8% vs. 18.4%, *p* = 0.076)	All bleeding events (11.2% vs. 12.7%, *p* = 0.82)
Patients with indication(s) for anticoagulation					
ENVISAGE TAVI AF (49), 2021 (*n* = 1,426)	Edoxaban 60 or 30 mg	VKA	18 months	Composite of all-cause death, MI, ischemic stroke, systemic thromboembolism, valve thrombosis, or major bleeding (17.3% vs. 16.5%, *p* = 0.01 for noninferiority)	Major bleeding (9.7% vs. 7.0%, *p* = 0.93 for non-inferiority)
ATLANTIS stratum 1 (47), 2022 (*n* = 451)	Apixaban 5 mg bid	VKA	1 year	Composite of death, MI, stroke, or TIA, non-CNS embolism, pulmonary embolism, intracardiac or valve thrombosis, DVT, and life-threatening, disabling, or major bleeding (22.0% vs. 21.9%, *p* = NS)	Life-threatening, disabling, or major bleeding (0.9% vs. 1.3%, *p* = NS)
POPular TAVI cohort B (52), 2020 (*n* = 331)	OAC (VKA or DOAC)	OAC + clopidogrel	1 year	Two primary endpoints: all bleeding (minor, major, life-threatening, or disabling) (21.7% vs. 34.6%, *p* = 0.01); non–procedure-related bleeding (21.7% vs. 34.0%, *p* = 0.02)	Primary endpoints

4D, 4-dimensional; AF, atrial fibrillation; CNS, central nervous system; CT, computed tomography; DVT, deep vein thrombosis; MI, myocardial infarction; NS, not significant; TAVI, transcatheter aortic valve implantation; TAVR, transcatheter aortic valve replacement; TIA, transient ischemic attack; VKA, vitamin K antagonist.

The POPular TAVI (Antiplatelet Therapy for Patients Undergoing Transcatheter Aortic Valve Implantation) trial also compared SAPT (aspirin) and DAPT (aspirin plus clopidogrel) (cohort A) and found that DAPT administered for 3 months did not reduce post-TAVR ischemic events but significantly increased the risk of all bleeding (26.6% vs. 15.1%, *p* = 0.001) and non–procedure-related bleeding (24.9% vs. 15.1%, *p* = 0.005) at the 12-month follow-up (39). Several meta-analyses confirmed that DAPT increases the risk of bleeding events without reducing thromboembolism or mortality rates compared with SAPT (40–45).

#### 4.1.2 Direct oral anticoagulants vs. antiplatelet therapy

The GALILEO (Global Study Comparing a Rivaroxaban-based Antithrombotic Strategy to an Antiplatelet-based Strategy after Transcatheter Aortic Valve Replacement to Optimize Clinical Outcomes) trial compared rivaroxaban 10 mg (plus aspirin 75–100 mg for the first 3 months) with aspirin 75–100 mg (plus clopidogrel 75 mg for the first 3 months) but was terminated prematurely because of higher rates of thromboembolic complications, bleeding, and mortality in the rivaroxaban group than in the antiplatelet therapy group (46).

In stratum 2 of the ATLANTIS (Anti-Thrombotic Strategy to Lower All Cardiovascular and Neurologic Ischemic and Hemorrhagic Events after Trans-Aortic Valve Implantation for Aortic Stenosis) trial, patients with no indications for anticoagulation received apixaban or antiplatelet therapy with aspirin and/or clopidogrel (as SAPT or DAPT) (47). Apixaban provided no net clinical benefit over antiplatelet therapy. Rates of mortality and the composite outcome of death, any stroke or TIA, or systemic embolism were significantly higher in the apixaban group than in the antiplatelet group, although bleeding rates were similar between groups.

The ADAPT-TAVR (Anticoagulation Versus Dual Antiplatelet Therapy for Prevention of Leaflet Thrombosis and Cerebral Embolization After Transcatheter Aortic Valve Replacement) trial compared the effectiveness of edoxaban vs. DAPT (aspirin plus clopidogrel) for preventing leaflet thrombosis at 6 months post TAVR (48). The incidence of leaflet thrombosis was lower with edoxaban than with DAPT, although the difference between groups was not statistically significant. There were also no significant between-group differences with regard to deaths, thrombo-ischemic events, or bleeding events. No significant association was observed between the presence or extent of leaflet thrombosis and new cerebral lesions or changes in neurologic or neurocognitive function.

Based on these clinical trials, SAPT is currently considered the first-line post-TAVR therapy for patients with no indications for anticoagulation. DAPT may be appropriate for patients who recently underwent coronary stenting or other endovascular procedures. Nevertheless, it is currently unclear which antiplatelet agent is preferable for SAPT. Although a retrospective study reported that clopidogrel monotherapy was associated with a lower incidence of cardiovascular death after TAVR compared with aspirin monotherapy, data are limited regarding this issue. Future studies are required to determine the optimal antiplatelet regimen(s) after TAVR.

### 4.2 Patients with indications for anticoagulation

#### 4.2.1 Direct oral anticoagulants vs. vitamin K antagonists

In the ENVISAGE AF-TAVI (Edoxaban vs. Standard of Care and Their Effects on Clinical Outcomes in Patients Having Undergone Transcatheter Aortic Valve Implantation–Atrial Fibrillation) trial, edoxaban was compared to a vitamin K antagonist (VKA) in patients with AF after TAVR (Table 1) (49). Although the primary composite outcome of thromboembolic events was similar between groups, the incidence of major bleeding (mainly gastrointestinal bleeding) was higher with edoxaban than with a VKA. However, in stratum 1 (patients with indications for anticoagulation) of the ATLANTIS trial, no differences were observed for any of the outcomes between apixaban and a VKA (47). Furthermore, a meta-analysis of five studies including a total of 2,569 patients found no significant differences in all-cause mortality, major and/or life-threatening bleeding, or stroke between direct oral anticoagulants (DOACs) and VKAs in patients undergoing TAVI with concomitant indications for oral anticoagulation (50).

By contrast, a study from the Society of Thoracic Surgeons/American College of Cardiology Transcatheter Valve Therapy Registry (including a total of 21,131 patients) found that in patients with AF, DOAC use was associated with a comparable risk of stroke but a lower incidence of any bleeding, intracranial hemorrhage, or death at 1 year after TAVR compared with VKA therapy (51). Therefore, although DOACs and VKAs appear to have comparable efficacy for preventing stroke in this patient population, there are discrepancies in the literature regarding the relative bleeding risks of these anticoagulation regimens.

#### 4.2.2 Anticoagulation vs. antiplatelet therapy plus anticoagulation

In cohort B of the POPular TAVI trial, patients with an indication for long-term anticoagulation (approximately 95% of whom had AF) received either an oral anticoagulant (OAC) alone (either a VKA or a DOAC) or a combination of an OAC plus clopidogrel after TAVR (52). At the 12-month follow-up, major bleeding as defined by VARC-2 was observed less frequently in the OAC alone group than in the OAC plus clopidogrel group. Two meta-analyses of patients requiring long-term anticoagulation also demonstrated that the risk of major and life-threatening bleeding was lower with an OAC regimen than with an OAC plus an antiplatelet agent, without affecting stroke rates (44, 53).

### 4.3 Antithrombotic therapy for the prevention and treatment of leaflet thrombosis

The LRT 2.0 (Strategies to Prevent Transcatheter Heart Valve Dysfunction in Low Risk Transcatheter Aortic Valve Replacement) trial was the first randomized trial to compare aspirin monotherapy vs. warfarin plus aspirin for the prevention of bioprosthetic valve dysfunction at 30 days after TAVR in low-risk patients (54). The rate of hypoattenuated leaflet thickening was 16.3% for aspirin and 4.7% for warfarin plus aspirin [*p* = 0.07; odds ratio, 4.0 (95% confidence interval, 0.8–20.0)]. There was no excess bleeding at 30 days in the patients who received warfarin and aspirin.

A substudy of the GALILEO trial showed that rivaroxaban was more effective than antiplatelet therapy for preventing subclinical leaflet thrombosis (detected on 4D-CT) at the 3-month follow-up after TAVR, but the increased risk of bleeding associated with rivaroxaban reported in the main GALILEO trial has limited its use in this setting (48, 55). In the ADAPT-TAVR trial comparing edoxaban vs. DAPT for the prevention of leaflet thrombosis at 6 months post TAVR, the incidence of leaflet thrombosis was lower with edoxaban, but the difference between groups was not statistically significant (48). Of note, subclinical leaflet thrombosis may appear and resolve multiple times with anticoagulation therapy, including DOACs or VKAs (18, 56). Given the poorly understood natural history and clinical implications of subclinical leaflet thrombosis, the need for preventive treatment remains unclear.

## 5 Guidelines

The latest guidelines from the European Society of Cardiology and the American College of Cardiology/American Heart Association provide specific recommendations for antithrombotic therapy after TAVR, tailored to patients with or without preexisting indications for anticoagulation (Table 2) (1, 2). Both sets of guidelines recommend aspirin monotherapy as the standard of care for most patients with no indications for long-term anticoagulation after TAVR. In patients requiring anticoagulation (such as those with AF), both guidelines favor oral anticoagulation alone and advise against routine combination therapy because of the increased risk of bleeding. Although the use of DAPT may still be considered in select patients, both guidelines have moved away from recommending it broadly after TAVR, prioritizing SAPT to mitigate bleeding complications.

**Table 2:** Current US and European guidelines for the management of antithrombotic therapies in patients undergoing transcatheter aortic valve replacement.

Class of recommendation	Level of evidence	Recommendations
ACC/AHA 2020 guidelines (1)		
2A	B	Aspirin 75–100 mg daily is reasonable in the absence of other indications for oral anticoagulants.
2B	B	For patients at low risk of bleeding, DAPT with aspirin 75–100 mg and clopidogrel 75 mg may be reasonable for 3–6 months after valve implantation.
2B	B	For patients at low risk of bleeding, anticoagulation with a VKA to achieve an INR of 2.5 may be reasonable for at least 3 months after valve implantation.
3	B	Treatment with low-dose rivaroxaban (10 mg daily) plus aspirin (75–100 mg) is contraindicated in the absence of other indications for oral anticoagulants.
ESC/EACTS 2021 guidelines (2)		
1	A	Lifelong SAPT is recommended after TAVI in patients with no baseline indication for OAC.
1	B	Lifelong OAC is recommended for patients who have other indications for OAC.
NA	NA	If recent coronary stenting (<3 months) and no concomitant indications for OAC, consider DAPT for 1–6 months and then SAPT.
		If recent coronary stenting (<3 months) and concomitant indication for OAC, continue lifelong OAC and consider SAPT for 1–6 months.
3	B	Routine use of OAC is not recommended after TAVI in patients with no baseline indication for OAC.

ACC, American College of Cardiology; AHA, American Heart Association; DAPT, dual antiplatelet therapy; EACTS, European Association for Cardio-Thoracic Surgery; ESC, European Society of Cardiology; INR, international normalized ratio; NA, not available; OAC, oral anticoagulation; SAPT, single antiplatelet therapy; VKA, vitamin K antagonist.

## 6 Ongoing trials and future directions

Several ongoing trials are investigating alternative antithrombotic strategies to refine post-TAVR management strategies. For example, in the ACLO-TAVR (Aspirin Versus Clopidogrel for Leaflet Thrombosis Prevention in Patients Undergoing Transcatheter Aortic Valve Replacement) trial, a planned total of 230 patients will first receive 4 weeks of DAPT (aspirin 100 mg and clopidogrel 75 mg) after TAVR and then be randomized to receive monotherapy with either aspirin or clopidogrel. The study will evaluate the incidence of leaflet thrombosis at 3 months post TAVI using cardiac CT and transthoracic echocardiography (NCT05493657). In the AVATAR (Anticoagulation Alone Versus Anticoagulation and Aspirin Following Transcatheter Aortic Valve Interventions) trial, OAC (DOAC or VKA) monotherapy is compared with OAC plus aspirin combination therapy post-TAVR (NCT02735902), and in the ACASA-TAVI (AntiCoagulation Versus AcetylSalicylic Acid After Transcatheter Aortic Valve Implantation) trial, a DOAC is compared with aspirin for the prevention of valve thrombosis after TAVR (NCT05035277). The POPular PAUSE TAVI (Periprocedural Continuation Versus Interruption of Oral Anticoagulant Drugs During Transcatheter Aortic Valve Implantation) trial compares the effects of pausing vs. maintaining OAC use perioperatively (NCT04437303). In the POPular ATLANTIS (Personalized, CT-guided Antithrombotic Therapy Versus Lifelong Single Antiplatelet Therapy to Reduce Thromboembolic and Bleeding Events in Non-atrial Fibrillation Patients After Transcatheter Aortic Valve Implantation) trial, variable antithrombotic treatment (based on the presence of thrombus on CT) is compared with lifelong SAPT following TAVR in patients with no indications for anticoagulation (NCT06168370). Furthermore, the NAPT (Non-antithrombotic Therapy After Transcatheter Aortic Valve Implantation) trial compares non-antithrombotic strategies with SAPT after TAVR in patients with a high risk of bleeding (NCT06007222) (57). Future clinical investigations should focus on a tailored approach based on both patient thromboembolic and bleeding risk profiles to improve the efficacy and safety of antithrombotic treatment post-TAVR.

## 7 Conclusion

The optimal antithrombotic regimen after TAVR is highly patient-specific, requiring careful balancing of the risks of thromboembolism and the risks of bleeding. For patients with no indications for anticoagulation, SAPT is favored over DAPT because of its lower risk of bleeding and comparable protection against ischemic/thromboembolic events. In patients requiring anticoagulation, monotherapy with a VKA or DOAC is sufficient, with the addition of antiplatelet therapy conferring no additional benefit but increasing the risk of bleeding.

Management of subclinical leaflet thrombosis and clinical valve thrombosis remains a complex issue, with ongoing studies expected to provide further clarity. Future research should focus on improving risk stratification and tailoring antithrombotic therapy to individual patient characteristics to optimize outcomes.
